# Molecular analysis of eight splicing variants in the hydroxymethylbilane synthase gene

**DOI:** 10.3389/fgene.2023.1291472

**Published:** 2023-11-20

**Authors:** Yi Ren, Jiajia Wang, Shuang Li, Jiajia Lei, Yunfeng Liu, Yan Wang, Fei Gao, Jianhong Wang, Jianhong Yin, Jing Yang

**Affiliations:** ^1^ Department of Endocrinology, The First Hospital of Shanxi Medical University, Taiyuan, Shanxi, China; ^2^ Department of Geriatrics, Shanxi Provincial People’s Hospital, Taiyuan, Shanxi, China; ^3^ Shanxi Medical University, Taiyuan, Shanxi, China; ^4^ Department of Neurology, Shanxi Cardiovascular Hospital, Taiyuan, Shanxi, China

**Keywords:** *hydroxymethylbilane synthase* gene, acute intermittent porphyria, splicing mutation, minigene, molecular analysis, porphyria

## Abstract

**Background:** Molecular genetic testing is the most sensitive and specific method to confirm acute intermittent porphyria (AIP), a rare autosomal dominant disease, caused by *Hydroxymethylbilane synthase (HMBS)* gene mutation. According to the Human Gene Mutation Database (HGMD), approximately 20% of the reported HMBS gene variants affect pre-RNA splicing. Thus, the ensuing challenge is how to decipher the pathogenicity of these splicing variants.

**Methods:** Using next-generation sequencing, we identified a novel heterozygous variant in the *HMBS* gene (c.160 + 5G>C) from a Chinese family with AIP. And, previously, seven HMBS variants (c.33 + 5G>A, c.88-16_88-4del, c.88-2A>G, c.161-1G>C, c.652-1G>A, c.772-2A>G and c.772-1G>C) have been reported to be linked with AIP. Herein, we performed a valid and novel in vitro minigene assay to analyze the pathogenicity of these eight splicing variants.

**Results:** By minigene assay in 293 T cell experiments, we demonstrated that all eight variants caused splicing defects in the pre-mRNA of the HMBS gene: c.160 + 5G>C (intron3p_141bp retention), c.33 + 5G>C(intron1p_91bp retention), c.88-16_88-4del and c.88-2A>G (Exon3p_15bp deletion), c.161-1G>C (Exon4p_18bp deletion), c.652-1G>A (Exon11p_1bp deletion), c.772-2A>G and c.772-1G>C (intron11q_104bp retention or Exon12p_4bp deletion).Encouragingly, the c.160 + 5G>C RNA sequencing from peripheral blood lymphocytes was consistent with the minigene assay result.

**Conclusion:** We have made a pioneering attempt to apply minigene *in vitro* validation to the HMBS gene to evaluate the splicing effect of eight variants, including a novel splice variant (c.160 + 5G>C). This study provides a molecular basis for future research on the pathogenesis and gene therapy of AIP.

## 1 Introduction

Acute intermittent porphyria (AIP, OMIM#176000), a rare autosomal dominant disease, is one of the most common types of hepatic porphyria (Besur et al., 2014). AIP is caused by defects in the *hydroxymethylbilane synthase* (*HMBS*) gene, resulting in a selected accumulation of heme precursors like 5-aminolevulinic acid (ALA) and porphobilinogen (PBG) in the heme biosynthesis pathway, which are associated with acute attacks of the disease (Puy et al., 2010). The clinical onset of AIP usually manifests at or after puberty (Innala et al., 2010) and is triggered by certain precipitating factors such as certain drugs, stress, fasting, and hormonal changes, which increase the body’s demand for heme (Roveri et al., 2014; Bissell et al., 2017). The clinical manifestations of AIP are complex and diverse, and there is substantial heterogeneity in severity, even within the same family (Puy et al., 2010; Szlendak et al., 2016). The main characteristics of the acute onset of this disease are abdominal pain with nausea and vomiting and neuropsychiatric symptoms, including disturbance of consciousness, epilepsy, hypertension, anxiety, and depression. Because abdominal pain is the most frequent and earliest symptom, it is considered to signal an acute attack (Ramanujam and Anderson, 2015; Kothadia et al., 2023). Of note, no single symptom is prevalent in patients with AIP. However, the penetrance of AIP is extremely low, and only 1% of heterozygous individuals carrying pathogenic mutations in the *HMBS* gene develop acute attacks, emphasizing the influence of modifying genes and environmental elements on major genes (Sassa, 2003; Chen et al., 2016). Furthermore, individuals with at least one acute attack in their lifetime are considered to have manifest AIP (MAIP), and individuals with latent AIP (LAIP) refer to patients with a high risk of progression to an acute exacerbation (Divecha et al., 2016). In addition, patients with LAIP have a high risk of liver cancer and renal failure (Pallet et al., 2015; Saberi et al., 2021).

We emphasize that the diagnosis of AIP cannot be completely based on the determination of urinary ALA, PBG, porphyrin, and *HMBS* activities because they lack sensitivity and specificity. For instance, uroporphyrin is elevated when liver function is impaired; the increase in urinary ALA and PBG levels was not detected in approximately 10% of patients in remission; and the activity levels of *HMBS* enzymes were significantly overlapped between patients with AIP and healthy relatives (Kauppinen and von und zu Fraunberg, 2002; Anderson et al., 2005). Moreover, in addition to timely treatment, the prognosis of this disease is attributed to accurate diagnosis and prevention of precipitating factors. Genetic screening can help confirm or exclude AIP, especially for patients with LAIP, which is an irreplaceable method to help high-risk family members make life decisions to prevent potentially life-threatening attacks (Whatley et al., 2009). Additionally, it can also help guide the classification of porphyrins, especially the identification of several subtypes of acute hepatic porphyria. Therefore, the molecular genetic detection of the *HMBS* gene is of great significance for the accurate diagnosis and prevention of this disease.

Interestingly, *HMBS* gene mutations were not identified in 5% of AIP families with clinical and biochemical characteristics due to the ignorance of splicing defects (Puy et al., 1997; Kauppinen and von und zu Fraunberg, 2002). The role of splicing variation in disease is significantly underestimated compared with synonym and nonsense variation (Lord and Baralle, 2021). It is estimated that pathogenic splicing variations account for 10%–50% of pathogenic mutations (Krawczak et al., 2007; Wang and Cooper, 2007; Sterne-Weiler et al., 2011; Vaz-Drago et al., 2017). The loss of function (LOF) of *HMBS* resulted in AIP. The *HMBS* gene, which comprises 15 exons, spans approximately 10 kilobase on 11q23.3 (Chretien et al., 1988). Splicing exon 1 to exon 3 leads to the expression of the housekeeping type of *HMBS*, which is widely expressed in all tissues and encodes 361 amino acids (*HMBS* is also called PBGD) (Gubin and Miller, 2001). So far, more than 500 *HMBS* gene variants have been reported in the Human Gene Mutation Database (HGMD). Among these, approximately 20% of the variants affect pre-RNA splicing (Zhang et al., 2021), most of which are in the canonical splice sites. It is worth noting that recent studies have highlighted deep intron variation and changes outside typical splicing sites as the causes of monogenic disorders by influencing pre-RNA splicing (Jian et al., 2014; Vaz-Drago et al., 2017). In recent years, with the improvement of second-generation gene sequencing technology and the in-depth interpretation of mutations, growing variants have been discovered. The current challenge of deciphering variants of uncertain significance (VUS) has led to the necessity of conducting functional analysis on these variants to determine their pathogenicity.

At present, the auxiliary methods for pathogenicity analysis of splicing variation mainly include software prediction, sample RNA analysis, and *in vitro* experiment analysis (Lord and Baralle, 2021). The most direct way to verify whether a mutated splice site is pathogenic is to perform reverse transcription RNA analysis (RT-PCR) by extracting RNA from the associated tissues of patients with AIP. However, it is difficult to extract quality and a sufficient quantity of RNA from individuals (Singh and Cooper, 2006). Some bioinformatic analysis software programs can predict the influence of mutations on splicing, but usually the predictive tools have moderate specificity (60%–80%) and tend to predict excessively harmful effects (Li et al., 2017). Furthermore, minigene assays are the best alternative approach to identifying the effect of gene mutation sites on pre-mRNA sequence splicing (Cooper, 2005).

Herein, we identified a novel heterozygous variant in the *HMBS* gene (c.160 + 5G>C) from a Chinese family with AIP. Previously, seven additional variants (c.33 + 5G>A, c.88-16_88-4del, c.88-2A>G, c.161-1G>C, c.652-1G>A, c.772-2A>G, and c.772-1G>C) have been reported to be linked with AIP, but the specific molecular characteristics have not been described so far (Yang et al., 2008; Kong et al., 2013; Li et al., 2015). In this study, with the use of a valid and novel *in vitro* minigene assay, we demonstrated that all eight sites caused splicing defects in the pre-mRNA of the *HMBS* gene. Additionally, we performed RNA extraction from the blood of individuals carrying this novel variant (c.160 + 5G>C), and the RT-PCR results were consistent with the minigene assay results, which further demonstrated the reliability of our *in vitro* experiments.

## 2 Materials and methods

### 2.1 DNA analysis

Genomic DNA was extracted from peripheral blood lymphocytes of the subject using the TIANamp Blood DNA Kit (TIANGEN, DP348-03) according to the manufacturer’s instructions. One-drop OD-1000 was used to detect DNA quality. The DNA was sent to WeHealth Company for next-generation sequencing of all exons and intron–exon junctions of the *HMBS* gene (NM_000190.4). Primers were designed according to the aforementioned results, followed by PCR amplification (F: 5′-ACACGACGCTCTTCCGATCTCTCATGCCCAGATGGAAATT-3′; R: 5′-CCTTGGCACCCGAGAATTCCACGAGCAGGAAGACCAGAAAC-3′; the underlined bases represent universal primers), and then Sanger sequencing was used to verify the variant (F: 5′-ACACGACGCTCTTCCGATCT-3′;R: 5′-CCTTGGCACCCGAGAATTCCA-3′).

### 2.2 Construction and expression of the minigenes

**FIGURE 1:**
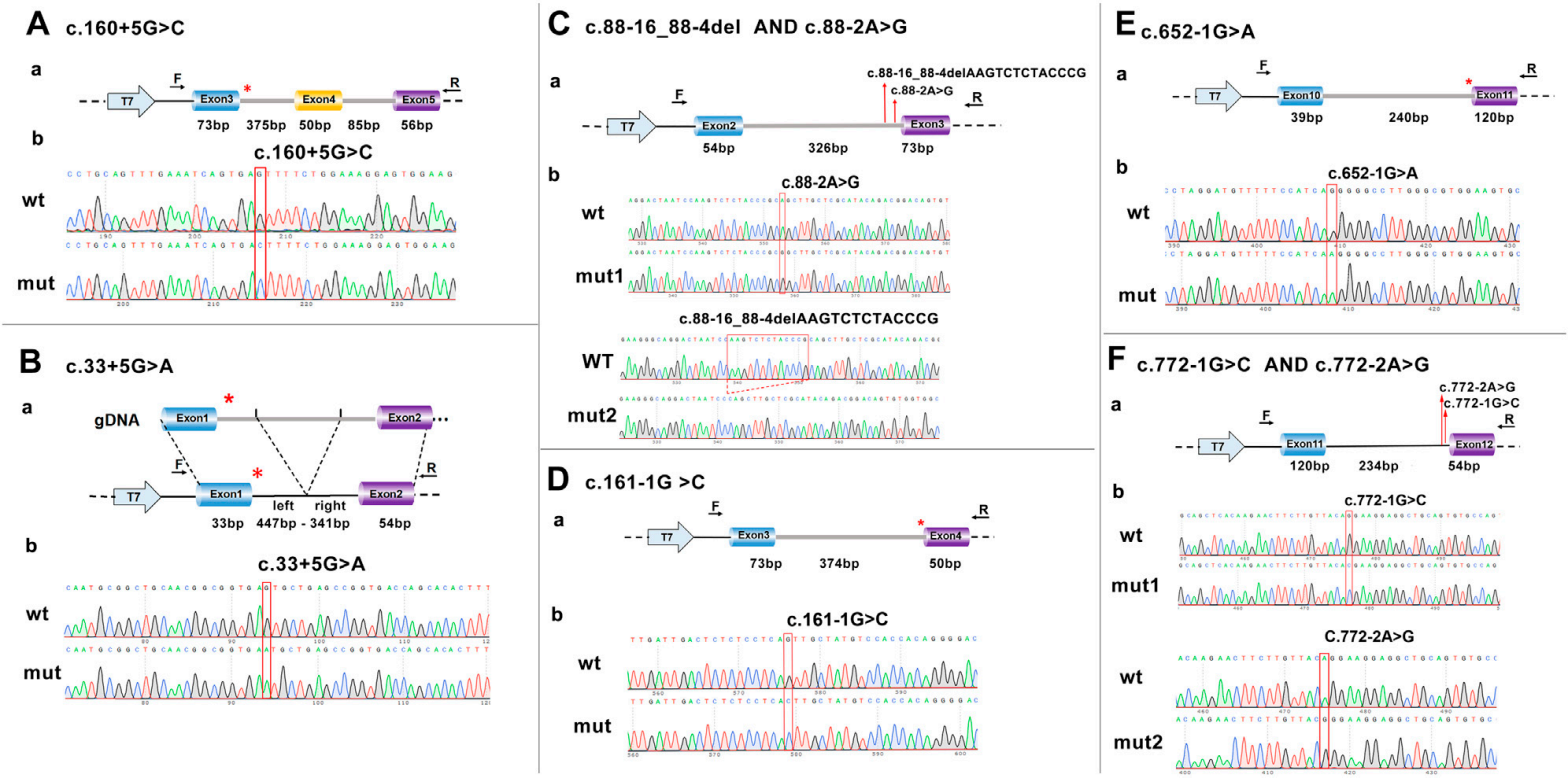
Schematic representation and sequencing of the minigene construction. **(A)** c.160 + 5G>C. **(B)** c.33 + 5G>A, in the c.33 + 5G>A variant minigene construction, intron 1 was shortened due to its excessive length. **(C)** c.88-16_88-4del AND c.88-2A>G. **(D)** c.160-1G>C. **(E)** c.652-1G>A. **(F)** c.772-1G>C AND c.772-2A>G. a, represents model diagram, b, represents gene sequencing. The red asterisk and arrow indicate the location of the variant. F, forward; R, reverse; T7, T7 promoter; wt, wild type; mut, mutant.

In order to explore whether these eight variants of the *HMBS* gene affect mRNA splicing, the eukaryotic expression vector pcDNA3.1 (+) was used. Using 100 ng normal human DNA as a template, the minigene wild-type fragment and the mutant fragment with two restriction enzyme cutting sites were amplified by PCR and overlap extension PCR with different primers for plasmid construction. See Supplementary Table S1 for specific information on primers and restriction enzyme cutting sites. Amplification conditions were set as follows: pre-denaturation at 94°C for 4 min, denaturation at 94°C for 30 s, annealing at 57°C for 30 s, extension at 72°C for 1 min of 30 cycles, and renaturation at 72°C for 10 min. After an enzyme digestion reaction at 37°C for 2 h, the target product and pcDNA3.1 (+) vector were recovered by agarose gel electrophoresis. After overnight ligation with T4 ligase, the recombinant vector was transformed into E. coli DH5α competent cells. After overnight culture, colony PCR was performed for verification, and positive clones were sequenced. Detailed information on the schematic diagram and sequencing of minigene construction for the eight variants is provided in Figure 1. The recombinant vector was transiently transfected into 293T cells according to the Lipofectamine 2000 specification method. Total RNA in the cells was extracted using TRIzol RNAiso Plus 24 h later, and reverse transcription PCR (RT-PCR) was performed using Hifair^®^ Ⅱ Reverse Transcriptase after the concentration was determined. The final cDNA was amplified using primers on both sides of the minigene fragment (F: 5′-CTA​GAG​AAC​CCA​CTG​CTT​AC-3′; R: 5′-TAG​AAG​GCA​CAG​TGA​GG-3′). Agarose gel electrophoresis was used to detect the size of transcript bands, and finally, Sanger sequencing was performed on the bands. The sequencing results were compared with the normal *HMBS* gene mRNA sequence.

### 2.3 RNA analysis

We performed *in vivo* analysis of the c.160 + 5G>C variant for the purpose of confirming the reliability of the minigene assay. The total RNA was extracted from the subject’s peripheral blood lymphocytes using the TRIzol reagent (Ambion, 15596026). A measure of 5°μg of total RNA was then reverse-transcribed into cDNA using the Hifair™ II 1st Strand cDNA Synthesis Kit (Yeason Biotechnology, 11121ES50). The reaction procedure was carried out at 25°C for 5 min, followed by 42°C for 60 min, and, finally, 85°C for 5 min. After PCR amplification of cDNA, Sanger sequencing was performed (amplification primer: F: 5′-ACA​CGA​CGC​TCT​TCC​GAT​CTGGA​AGA​AAA​CAG​CCC​AAA​GA-3′; R: 5′-CCT​TGG​CAC​CCG​AGA​ATT​CCAAA​GCC​AGG​AGG​AAG​CAC​AG-3′. Universal primers were used for sequencing: F: 5′-ACA​CGA​CGC​TCT​TCC​GAT​CT-3′; R: 5′- CCT​TGG​CAC​CCG​AGA​ATT​CCA-3′).

## 3 Results

### 3.1 Identification and molecular analysis of the variant c.160 + 5G>C

**FIGURE 2:**
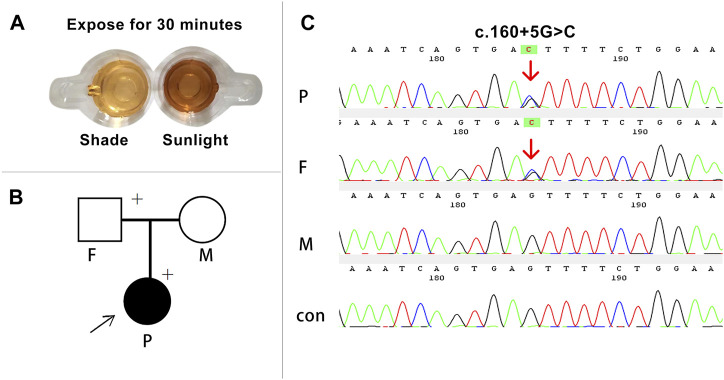
Clinical and molecular diagnosis of the proband. **(A)** The proband’s urine turned brown-red after 30 min of sun exposure. **(B)** The arrow indicates the proband, and the plus sign indicates the carrier of the variant gene. **(C)** Sanger sequencing of the family members and a control. Heterozygous variants c.160 + 5G>C (red arrow) were detected in the proband and her father. P, proband; F, father; M, mother; con, control.

The proband was a 26-year-old women of Han ethnicity. Her diagnosis of AIP was based on three acute attacks, clinical symptoms of abdominal pain, red urine after sun exposure, urine PBG (+), and lead-negative urine (Figure 2A). None of the patient’s family members had a history of similar diseases.

DNA Sanger sequencing of the proband and her family discovered a heterozygous variant in the *HMBS* gene (c.160 + 5G>C) (Figures 2B, C). The variant located at the non-classical splice donor site in intron 3 is not presented in the HGMD (HGMD, http://www.hgmd.org) or the 1000 Genome Project (1000G, http://www. 1000genomes.org), confirming that it is a novel splicing variant that has not been reported previously. The variant was also detected in her father and was not a *de novo* variant. The results predicted using Human Splicing Finder software (http://www.umd.be/HSF3) indicate alteration of the wild-type donor site, most probably affecting splicing (Table 1). According to the American College of Medical Genetics and Genomics (ACMG) guidelines (Richards et al., 2015), the variant c.160 + 5G>C was classified as VUS.

**TABLE 1:** Eight splice variants in the *HMBS* gene in this study.

	Variant	Location	Human splicing finder predictive analysis	Results of Minigene Assay	Predicted change in the amino acid	Reference
1	c.160+5G>C	Intron 3	Alteration of the WT donor site	141-bp retention at the 5'-end of intron 3	p.(Ile54Serfs*29)	This study
2	c.33+5G>A	Intron 1	Alteration of the WT donor site	91-bp retention at the 5'-end of intron 1	p.(Glu12Valfs*71)	Yang et al. (2008)
3	c.88-16_88−4del	Intron 2	Alteration of the WT acceptor site	15-bp deletion at the 5'-end of exon 3	p.(Leu30_Gln34del)	Yang et al. (2008)
4	c.88–2A>G	Intron 2	Alteration of the WT acceptor site	15-bp deletion at the 5'-end of exon 3	p.(Leu30_Gln34del)	Yang et al. (2008)
5	c.652−1G>A	Intron 10	Alteration of the WT acceptor site, most probably affecting splicing	1-bp deletion at the 5'-end of exon 11	p.(Ala219Profs*36)	Yang et al. (2008)
			Activation of an intronic cryptic acceptor site, leading to potential alteration of splicing			
6	c.772−1G>C	Intron 11	Alteration of the WT acceptor site	4-bp deletion at the 5'-end of exon 12	p.(Glu258Aspfs*23)	Yang et al. (2008)
				104-bp retention at the 3'-end of intron 11	p.(Gly2-−59Alafs*6)	
7	c.772–2A>G	Intron 11	Alteration of the WT acceptor site	4-bp deletion at the 5'-end of exon 12	p.(Glu258Aspfs*23)	Kong et al. (2013)
				104-bp retention at the 3'-end of intron 11	p.(Gly259Alafs*6)	
8	c.161−1G>C	Intron 3	Alteration of the WT acceptor site	18-bp deletion at the 5'-end of exon 4	p.(Ala54_Gly60delinsArg)	Li et al. (2015)

**FIGURE 3:**
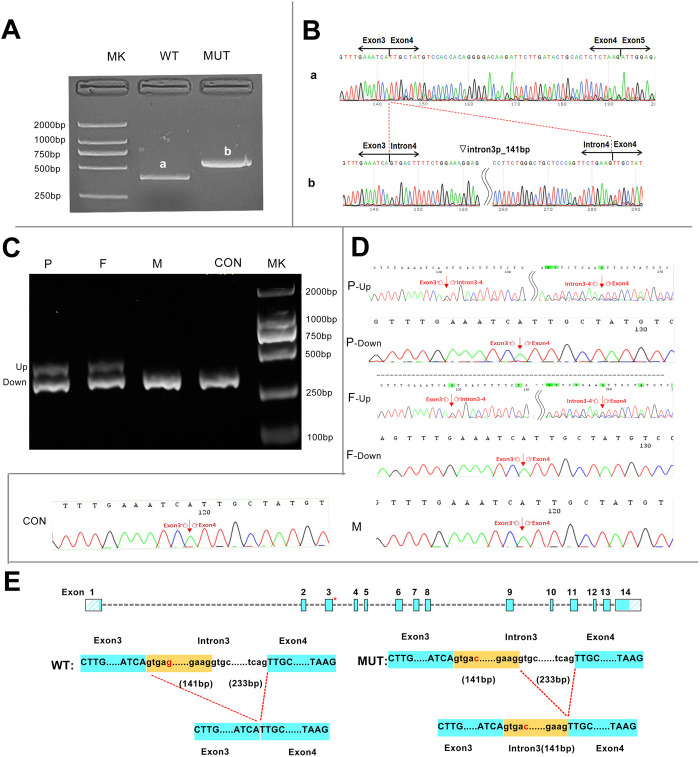
Transcript analyses for the c.160 + 5G>C variant were obtained from the minigene assay and *in vivo* experiments. **(A)** Agarose gel results of the minigene assay for c.160 + 5G>C showed that the mutant clone had a larger band than the wild-type clone, and the two bands were named a and b, respectively. **(B)** Sanger sequencing of bands a and b. Band a was the normally spliced transcript, and band b was an aberrant transcript with a 141-bp retention at the 5′-end of intron 3. Inverted triangle, retention; p, at the 5’-end. **(C)** *In vivo* agarose gel electrophoresis for c.160 + 5G>C showed that all family members and the control had a band of same size named the DOWN band, and the proband and her father had an additional band of larger molecular weight named the UP band. **(D)** Sanger sequencing of the *in vivo* results showed that the Down band was the normally spliced transcript, and band b was an aberrant transcript with a 141-bp retention at the 5-end of intron 3.**(E)** Schematic representation of aberrant splicing caused by c.160 + 5G>C. The red asterisk indicates the position of c.160 + 5G>C on the *HMBS* gene. MK, DNA molecular weight marker; P, proband; F, father; M, mother; CON, control; WT, wild type; MUT, mutant.

We successfully constructed the recombinant expression vectors of wild-type and c.160 + 5G>C mutant. After transfection into 293T cells, RT-PCR products were checked by agarose gel electrophoresis and sent for Sanger sequencing. Compared with the wild-type, a larger band was present in the mutant clone in agarose gel electrophoresis (Figure 3A). Sanger sequencing analysis showed that the novel mutation c.160 + 5G>C caused a 141-bp retention at the 5′-end of intron 3 (Figure 3B).

In order to further investigate the reliability of the minigene assay, *in vivo* experiments were performed. The RT-PCR products of lymphocyte RNA from the three family members all had the band of same size as that of the healthy controls. However, the proband and her father had a remaining band with a larger molecular weight on agarose gel electrophoresis (Figure 3C). The up band denotes an abnormal transcript with 141 bp retained at the 5′-end of intron 3 containing the mutation site, which is consistent with the product of the mutation clone in the minigene assay. In addition, the down band denotes a transcript with normal splicing of exons 3 and 4 (Figures 3D, E).

### 3.2 Seven variants affect splicing in the minigene assay

In silico tools using HSF indicated that these seven variants in the *HMBS* gene had a great probability of causing splicing defects (Table 1). A minigene assay was performed to validate the pathogenicity of these seven variants (five canonical splice sites and two exon–intron boundary sites).

**FIGURE 4:**
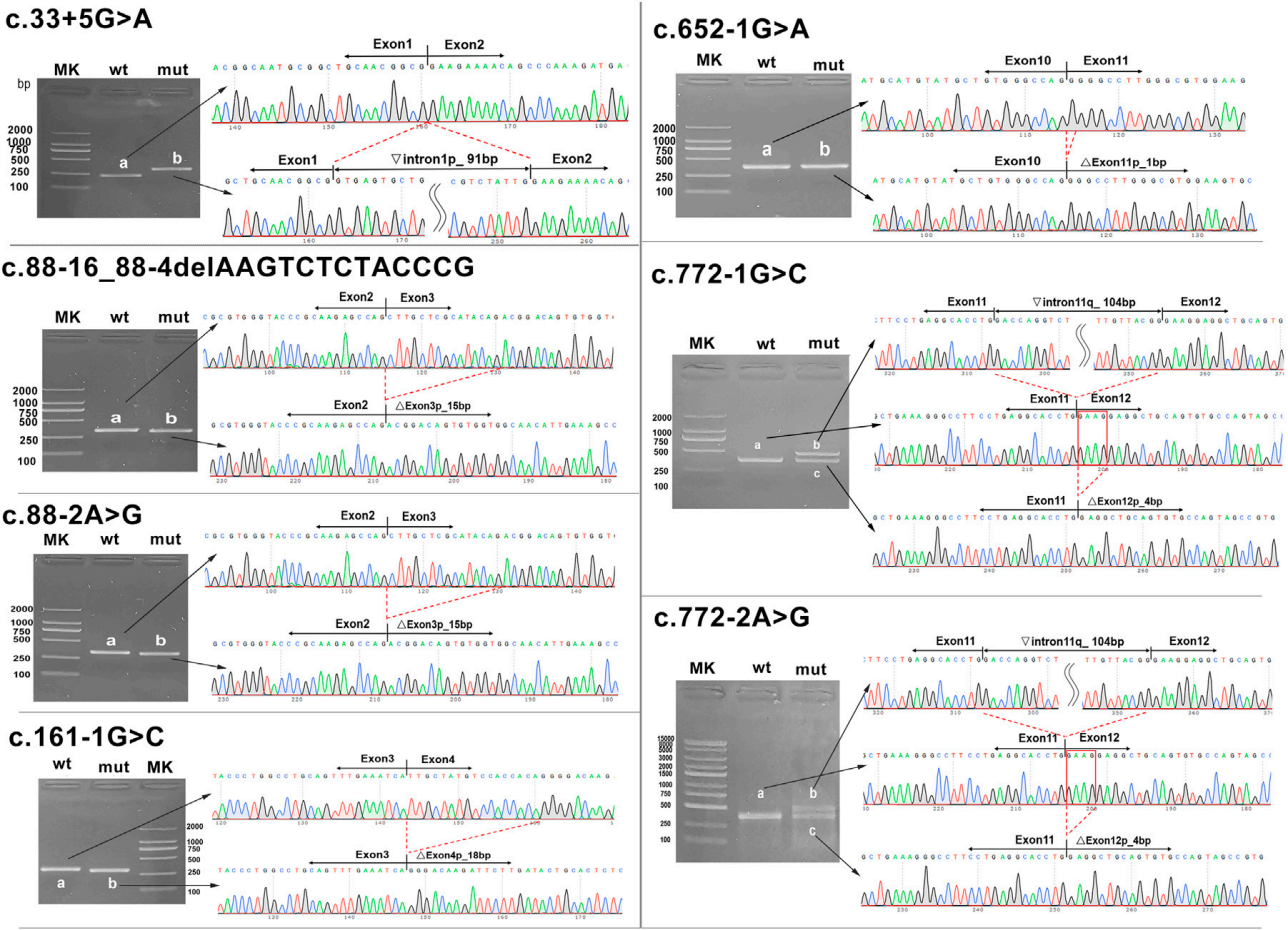
Transcript analyses for seven *HMBS* gene variants. Agarose gel electrophoresis and Sanger sequencing of RT-PCR products were obtained after transfection of the recombinant vector with the mutation site into 293T cells. The red line frame indicates aberrant base sequences in mature transcripts caused by variants. MK, DNA molecular weight marker; wt, wild type; mut, mutant. Inverted triangle, retention; Vertical triangle, deletion; p, at the 5’-end; q, at the 3’-end.

In two variants located in the exon–intron boundary region, the c.88-16_88-4del variant caused a 15-bp deletion at the 5′-end of exon 3 due to the loss of 13 bp of the normal sequence, while a single-nucleotide substitution of the c.33 + 5G>C variant caused a 91-bp retention at the 5′-end of intron 1. Five variants replacing one nucleotide of the classical splice site all resulted in abnormal transcripts with partial deletion of adjacent exons (c.88-2A>G, c.161-1G>C, c.652-1G>A, c.772-1G>C, and c.772-2A>G), but in two cases, multiple transcripts were expressed. Variants c.772-1G>C and c.772-2A>G had the same effect on splicing and produced two different sizes of transcripts in 293T cell expression: a 4-bp deletion at the 5′-end of exon 12 and a 104-bp retention at the 3′-end of intron 11, suggesting that there are multiple mechanisms coexisting in the process of altered RNA maturation (Figure 4). The minigene assay confirmed that seven *HMBS* gene variants were pathogenic as they caused splicing defects in pre-mRNA.

## 4 Discussion

Although the rules and languages of splicing have been intensively studied over the past 40 years, our understanding of the specific mechanisms of splicing is still inadequate. The study of specific aberrant splicing results caused by variants is not only a necessary condition to link variants with diseases but also provides evidence-based information for the exploration of aberrant splicing mechanisms.

In this study, we identified and characterized one novel and seven previously reported variants (five classical splice sites and three intronic splice sites) at the molecular level, all of which were confirmed to cause aberrant splicing and produce aberrant transcripts. The resulting mRNA effect was dominated by partial deletion of exons, followed by intron retention. Previously reported exon skipping was the most common splicing type, and it is possible that this type is also present in the eight mutations, although it was not observed in our study (Nakai and Sakamoto, 1994; Pros et al., 2008).

According to the classification of splice variants by Wimmer et al., the five splicing variants (c.88-2A>G, c.161-1G>C, c.652-1G>A, c.772-2A>G, and c.772-1G>C) located in the classical region can be classified as type IV splice variants causing intron retention or exon loss by activating the nearby cryptic receptor splice site (Wimmer et al., 2007; Anna and Monika, 2018). It is worth noting that there is a highly conserved nucleotide consensus sequence at the splicing site of eukaryotic pre-mRNA. When the last nucleotide guanine (G) of the acceptor splice site is replaced by adenine (A), A recombines with the first nucleotide G of the GU at the 3'-end of the adjacent exon, creating a new splice recognition site. It can be predicted that when G>A occurs in the canonical-1 splice site, a 1-bp deletion at the 3′-end of the downstream exon will be generated, such as the variant c.652-1G>A. Although this mutation transcripts only a small 1-bp deletion at the start of exon 11, the *HMBS* enzyme activity was significantly decreased due to an in-frame frameshift, leading to a truncated protein (p. (Ala219Profs*36)) (Lin et al., 2018). Variants c.772-1G>C and c.772-2A>G produced two identical abnormal transcripts, which caused premature termination of translation by protein prediction analysis (p. (Glu258Aspfs*23) and p. (Gly259Alafs*6)). The mechanism that causes the decrease in enzyme activity may be the inability of the cofactor DPM to covalently bind to C261 to start the catalytic reaction (Jordan and Warren, 1987; Morán-Jiménez et al., 2020).

In the case of the variant c.33 + 5G>A, disruption of the splicing consensus sequence leads to activation of a cryptic donor splice site at nucleotide 91 at the 5′-end of intron 1, resulting in a premature termination codon (PTC) in the coding sequence. Therefore, the appearance of a truncated polypeptide chain containing 83 amino acids caused a significant decrease in enzyme activity [38]. Splicing defects caused by variants at the junction of exon 1 and intron 1 have been confirmed in many studies, and four variants with nucleotide substitutions at positions c.33 + 1 to +5 resulted in the activation of a cryptic donor splice site of 67 bp at the 5′-end of intron 1 (Mustajoki et al., 1998; Puy et al., 1998; Granata et al., 2018). The choice of flexibility of the cryptic splice site accounts for the difference in this result, with strength and distance from the classical splice site playing a major role in determining the activation of the cryptic splice site (Ramalho et al., 2003; Dawes et al., 2022). Interestingly, both the variants c.88-16_88-4del and c.88-2A>G produced an aberrant transcript with a 15-bp deletion in the head of exon 3, resulting in a defective protein with a deletion of positions 30–34 amino acids. At the same time, the previously reported variant c.88-1G>C also produced the same transcript. Protein structure analysis manifested that the five missing amino acids were close to the active site regions Arg26 and Ser28, which caused the cofactor DPM to fail to bind to the gap of domains 1 and 2, and the formation of the catalytic active center of the *HMBS* enzyme was blocked, thereby reducing the enzyme activity, as shown by the detection of *HMBS* enzyme activity (Zhang et al., 2021). We identified a novel variant c.160 + 5G>C located in the non-canonical splicing region. The results of *in vivo* reverse transcription assays were completely consistent with those of minigene assays, which directly confirmed the accuracy of minigene assays in assessing whether *HMBS* gene variants affect splicing. According to ACMG guidelines, PS3 evidence can be provided for the variant c.160 + 5G>C (Richards et al., 2015).

Nowadays, with the improvement of next-generation gene sequencing technology and the in-depth interpretation of mutations, it has been found that the missed rate of splicing mutations is higher than that of nonsense mutations and missense mutations (Padgett, 2012). At present, the molecular epidemiology of AIP has been widely studied. Among the more than 500 *HMBS* gene variants reported by HGMD, 108 of them are variants affecting splicing (Zhang et al., 2021). The proportion of *HMBS* gene splicing defects was consistent with that reported in other countries. Splicing variants accounted for 26% of reports from Argentina, all of which were located in classical regions (Cerbino et al., 2015). Splicing variants were found in 12 of 32 Spanish porphyria genes (Morán-Jiménez et al., 2020). However, this proportion may be higher because most missense and non-variant variants have not been noted for splicing-related functional evaluation. Another report of *HMBS* mutations in Spain confirmed that c.771 + 58C>T is a genetic polymorphism (Guillén-Navarro et al., 2004). Among the 40 mutation sites reported by Brenden Chen et al., eight of them were splicing mutation sites. It is worth mentioning that of the eight splicing mutation sites, the mutation site c.612G>T causes changes in *HMBS* transcripts (the missense variant c.612G>T p. (Gln204His) is located on the last nucleotide of exon 10 and results in a nine-nucleotide deletion at the 3′-end of exon 10) (Chen et al., 2019). Therefore, in view of the diversity of *HMBS* mutation sites, the high frequency of gene polymorphism, and the complexity of the splicing mechanism, it is important to evaluate the continuous detection of splicing mutation sites of unknown pathogenicity, especially in the borderline and deep introns.

## 5 Conclusion

We made a pioneering attempt to apply minigene *in vitro* validation to the *HMBS* gene to evaluate the splicing effect of eight variants. These variants produce abnormal transcripts, indicating their pathogenicity at the RNA level, which provides PS3 evidence according to the American College of Medical Genetics and Genomics (ACMG) guidelines. We further performed an *in vivo* analysis of the novel splice variant (c.160 + 5G>C), and the results were consistent with those of the minigene assay, confirming the reliability of this method. This study provides a molecular basis for future exploration of the pathogenesis and gene therapy of AIP. Moreover, we highlight the importance of assessing the pathogenicity of non-canonical splice sequence variants in disease diagnosis.

## Data Availability

The original contributions presented in the study are included in the article/Supplementary Material; further inquiries can be directed to the corresponding authors.
