# Supplementary material for: Molecular analysis of eight splicing variants in the hydroxymethylbilane synthase gene
Source: Front Genet. 2023 Nov 20;14:1291472. doi: 10.3389/fgene.2023.1291472 (PMC10698471; doi:10.3389/fgene.2023.1291472)
Supplement: Supplementary file 1 [file Table1.docx]

**Supplementary Information**

**Molecular analysis of eight splicing variants in Hydroxymethylbilane synthase gene**

Yi Ren Jiajia Wang Shuang Li Jiajia Lei Yunfeng Liu Yan Wang Fei Gao Jianhong Wang Jianhong Yin  Jing Yang

Corresponding author : renyi_0_0@163.com(Yi.Ren.) ;

yangjlm@126.com(Jing.Yang.)

**Table S1 Primers used in minigene construction**

| variant | NO | Primer name | Role | primer sequences(5’→3’) |
| --- | --- | --- | --- | --- |
| c.160+5G>C^a^ | 1 | HMBS-2911-F | Primer 1 | ctcccactgacaactgcctt |
|  | 2 | HMBS-3221-F | Primer 2 | GAAGAAAACAGCCCAAAGA |
|  | 3 | HMBS-4396-R | Primer 3 | tgaagcctgaaaaaacccac |
|  | 4 | HMBS-4706-R | Primer 4 | AAGCCAGGAGGAAGCACAG |
|  | 5 | HMBS-KpnI-F |  | GCTT*GGTACC*atgCTTGCTCGCATACAGACG |
|  | 6 | HMBS-BamHI-R |  | TAGT*GGATCC*tTCATTCTTCTCCAGGGCATG |
| c.33+5G>A^b^ | 1 | HMBS-591-F | Primer 1 | gagttcgagactagcctgggc |
|  | 2 | HMBS-885-F | Primer 2 | cactacttgctcatgtcacag |
|  | 3 | HMBS-4715-R | Primer 3 | cacggctcagactctttagactg |
|  | 4 | HMBS-5011-R | Primer 4 | ctaatccaggtagtcagagctgg |
|  | 5 | HMBS-WT-HindIII-F | Primer A1 | ACTT*AAGCTT*ATGTCTGGTAACGGCAATGCGG |
|  | 6 | HMBS-MUT-HindIII-F | Primer A1 | ACTT*AAGCTT*ATGTCTGGTAACGGCAATGCGGCTGCAACGGCGgtgaAtgctgagccggtga |
|  | 7 | HMBS-EcoRI-R | Primer B2 | TGCA*GAATTC*CTGGCTCTTGCGGGTACCCACG |
|  | 8 | HMBS-lap-F | Primer B1 | gtatcagccaagcctatctctttacccacc |
|  | 9 | HMBS-lap-R | Primer A2 | ggtgggtaaagagataggcttggctgatac |
| c.88-16_88-4delAAGTCTCTACCCG | 1 | HMBS-3724-F | Primer 1 | cagtccagccagattaaggg |
|  | 2 | HMBS-4066-F | Primer 2 | cccactgacaactgccttggtc |
|  | 3 | HMBS-5011-R | Primer 3 | ctaatccaggtagtcagagctgg |
|  | 4 | HMBS-5595-R | Primer 4 | gcagtccaggcagccagatggc |
|  | 5 | HMBS-88-4-MUT-F | Primer B1 | gggcaggactaatcccagCTTGCTCGCATAC |
|  | 6 | HMBS-88-4-MUT-R | Primer A2 | GTATGCGAGCAAGctgggattagtcctgccc |
|  | 7 | HMBS-88-HindIII-F | Primer A1 | T*AAGCTT*ATGGAAGAAAACAGCCCAAAGAT |
|  | 8 | HMBS-88-BamHI-R | Primer B2 | GT*GGATCC*AATGATTTCAAACTGCAGGCCA |
| c.88-2A>G | 1 | HMBS-3724-F | Primer 1 | cagtccagccagattaaggg |
|  | 2 | HMBS-4066-F | Primer 2 | cccactgacaactgccttggtc |
|  | 3 | HMBS-5011-R | Primer 3 | ctaatccaggtagtcagagctgg |
|  | 4 | HMBS-5595-R | Primer 4 | gcagtccaggcagccagatggc |
|  | 5 | HMBS-88-2-MUT-F | Primer B1 | caagtctctacccgcGgCTTGCTCGCATACAG |
|  | 6 | HMBS-88-2-MUT-R | Primer A2 | CTGTATGCGAGCAAGcCgcgggtagagacttg |
|  | 7 | HMBS-88-HindIII-F | Primer A1 | T*AAGCTT*ATGGAAGAAAACAGCCCAAAGAT |
|  | 8 | HMBS-88-BamHI-R | Primer B2 | GT*GGATCC*AATGATTTCAAACTGCAGGCCA |
| c.161-1G>C | 1 | HMBS-3724-F | Primer 1 | cagtccagccagattaaggg |
|  | 2 | HMBS-4066-F | Primer 2 | cccactgacaactgccttggtc |
|  | 3 | HMBS-5595-R | Primer 3 | gcagtccaggcagccagatggc |
|  | 4 | HMBS-5912-R | Primer 4 | ctgcccaagccccttacttg |
|  | 5 | HMBS-161-MUT-F | Primer B1 | ttgactctctcctcaCTTGCTATGTCCACCA |
|  | 6 | HMBS-161-MUT-R | Primer A2 | TGGTGGACATAGCAAGtgaggagagagtcaa |
|  | 7 | HMBS-161-Kpn1-F | Primer A1 | gctt*ggtacc*atgCTTGCTCGCATACAGACGGAC |
|  | 8 | HMBS-161-BamHI-R | Primer B2 | CGGT*GGATCC*CTTAGAGAGTGCAGTATCAAG |
| c.652-1G>A | 1 | HMBS-7688-F | Primer 1 | cctttctgccttacagtcatccc |
|  | 2 | HMBS-7979-F | Primer 2 | ggatgtaaccctattgtaag |
|  | 3 | HMBS-9340-R | Primer 3 | CAAGTTGGCCAGGCTGATGCC |
|  | 4 | HMBS-9608-R | Primer 4 | CTCTAAAGAGATGAAGCCCCCA |
|  | 5 | HMBS-652-MUT-F | Primer B1 | gatgtttttccatcaAGGGGCCTTGGGCGTG |
|  | 6 | HMBS-652-MUT-R | Primer A2 | CACGCCCAAGGCCCCTtgatggaaaaacatc |
|  | 7 | HMBS-652-Kpn1-F | Primer A1 | gctt*ggtacc*atgATCCTGCACCCTGAGGAATG |
|  | 8 | HMBS-652-BamHI-R | Primer B2 | CGGT*GGATCC*CAGGTGCCTCAGGAAGGCCC |
| c.772-1G>C | 1 | HMBS-7688-F | Primer 1 | cctttctgccttacagtcatccc |
|  | 2 | HMBS-7979-F | Primer 2 | ggatgtaaccctattgtaag |
|  | 3 | HMBS-9340-R | Primer 3 | CAAGTTGGCCAGGCTGATGCC |
|  | 4 | HMBS-9608-R | Primer 4 | CTCTAAAGAGATGAAGCCCCCA |
|  | 5 | HMBS-772-1-MUT-F | Primer B1 | gaacttcttgttacaCGAAGGAGGCTGCAGT |
|  | 6 | HMBS-772-1-MUT-R | Primer A2 | ACTGCAGCCTCCTTCGtgtaacaagaagttc |
|  | 7 | HMBS-772-HindIII-F | Primer A1 | ACTT*AAGCTT*ATGGGGGCCTTGGGCGTGGAAGT |
|  | 8 | HMBS-772-BamHI-R | Primer B2 | TAGT*GGATCC*TTGCCCATCCTTCATAGCTG |
| c.772-2A>G | 1 | HMBS-7688-F | Primer 1 | cctttctgccttacagtcatccc |
|  | 2 | HMBS-7979-F | Primer 2 | ggatgtaaccctattgtaag |
|  | 3 | HMBS-9340-R | Primer 3 | CAAGTTGGCCAGGCTGATGCC |
|  | 4 | HMBS-9608-R | Primer 4 | CTCTAAAGAGATGAAGCCCCCA |
|  | 5 | HMBS-772-2-MUT-F | Primer B1 | agaacttcttgttacGgGAAGGAGGCTGCAG |
|  | 6 | HMBS-772-2-MUT-R | Primer A2 | CTGCAGCCTCCTTCcCgtaacaagaagttct |
|  | 7 | HMBS-772-HindIII-F | Primer A1 | ACTT*AAGCTT*ATGGGGGCCTTGGGCGTGGAAGT |
|  | 8 | HMBS-772-BamHI-R | Primer B2 | TAGT*GGATC*CTTGCCCATCCTTCATAGCTG |

Note:

**Superior characters**:a)The mutant fragment of c.160+5G>C is from patients’ blood sample. b) The wildtype fragment of c.33+5G>A is from the result of overlap PCR for truncated intron1.

**In Role column**:1)Primers 1~4 are designed for Nested PCR. Round 1：primer 1 designates Forward Outer Primer, and primer 4 designates Reverse Outer Primer . Round 2 ：primer 2 designates Forward Inner Primer, and primer 3 designates Reverse Inner Primer. 2)Primers A1,A2,B1 and B2 are designed for Overlap PCR. Primer A1 and Primer A2 are used to amplify product A. Primer B1 and Primer B2 are used to amplify product B. Primer A1 and Primer B2 are used to amplify the target product(A+B).

**In primer sequences column**: The black lowercase letters represent intron sequences; The black uppercase letters represent exon sequences. The underline letters indicates the restriction site. The blue letters represent the randomly inserted bases. The red letters represent the bases of variation.

**In Primer name column**: Abbreviations: F: forward primer; R: reversed primer.
